# MicroRNA Assisted Gene Regulation in Colorectal Cancer

**DOI:** 10.3390/ijms20194899

**Published:** 2019-10-03

**Authors:** Adewale O. Fadaka, Ashley Pretorius, Ashwil Klein

**Affiliations:** Department of Biotechnology, Faculty of Natural Sciences, University of the Western Cape, Private Bag X17, Bellville, 7535 Cape Town, South Africa; aspretorius@uwc.ac.za (A.P.); aklein@uwc.ac.za (A.K.)

**Keywords:** molecular interaction, microRNA, *in silico* prediction, target gene, gene expression, silencing, colorectal cancer

## Abstract

Colorectal cancer (CRC) is the second-leading cause of cancer death and a major public health problem. Nearly 80% CRC cases are diagnosed after the disease have metastasized and are often too advanced for treatment. Small non-coding RNA guides argonaute protein to their specific target for regulation as the sole of RNA induced silencing complex for gene silencing. These non-coding RNA for example microRNA, are thought to play a key role in affecting the efficiency of gene regulation in cancer, especially CRC. Understanding the mechanism at the molecular level could lead to improved diagnosis, treatment, and management decisions for CRC. The study aimed to predict the molecular mechanism of gene regulation based microRNA-mRNA duplex as a lead in the silencing mechanism. Five candidate microRNAs were identified through the in silico approach. The MicroRNA target prediction and subsequent correlation, and prioritization were performed using miRTarBase, gbCRC and CoReCG, and DAVID databases respectively. Protein selection and preparation were carried out using PDB and Schrödinger suits. The molecular docking analysis was performed using PATCHDOCK webserver and visualized by discovery studio visualizer. The results of the study reveal that the candidate microRNAs have strong binding affinity towards their targets suggesting a crucial factor in the silencing mechanism. Furthermore, the molecular docking of the receptor to both the microRNA and microRNA-mRNA duplex were analyzed computationally to understand their interaction at the molecular level. Conclusively, the study provides an explanation for understanding the microRNAs-based gene regulation (silencing mechanism) in CRC.

## 1. Introduction

Colorectal cancer (CRC) is considered as one of the most threatening diseases due to its incidence and mortality rate worldwide [1], and the most frequent cancers in western world [2]. Over 1.2 million individuals are diagnosed with this disease yearly, and over 600,000 mortalities are recorded [3]. Although the activation and inactivation of oncogenes and tumor suppressor genes respectively are known to be involved in CRC development at molecular level [4], the molecular mechanisms that lead to the development and progression of CRC remain unclear.

Despite the advances in the diagnosis, treatment, and management of patients with CRC, it is still a major public health problem globally [5]. Therefore, it is imperative to elucidate the mechanism of gene silencing in the tumorigenesis of CRC for better understanding. 

The interactions between protein and nucleic acids play essential roles in various cellular and biological processes, including DNA replication, RNA transcription, the translation of polypeptides, RNA splicing, and the degradation of nucleic acids [6,7]. The errors in receptor-nucleic acid interactions are implicated in a number of diseases, ranging from neurological disorders to cancer [8]. RNA binding proteins are mediators of RNA silencing processes, such as pathways in microRNA and RNA interference. Argonaute, a unique member of this family [9], forms the functional core of the RNA induced silencing complex (RISC) in humans [10]. The RISC complexed with AGO employs small molecules, such as microRNA, as a guide for target recognition and silencing through translational repression and/or degradation [11]. 

MicroRNAs are small non-coding RNAs with 18–22 nucleotide sequences possessing regulatory roles in both plants and animals. These non-coding RNAs are involved in different cellular processes [12,13,14,15,16] including human diseases [17], such as colorectal carcinogenesis [18]. Additionally, experiments revealed that these RNAs can act as oncomiR [19,20] and/or tumor suppressor microRNAs [21] and their differential expression between normal and abnormal tissue have been exploited in the diagnosis, treatment, and management of CRC [22]. The epigenetic regulation of gene expression at a transcriptional or post-transcriptional level is important as a mechanism of gene silencing. 

Various experimental approaches [23] have been put forward to study the mechanism by which cancer genes are repressed, inactivated or silenced to prevent carcinogenesis, progression or metastasis of the involved gene. Recently, the microRNA binding proteins became a focal point in cancer research due to their involvement in microRNAs deregulation [24,25]. Argonaute utilizes microRNAs and RNA interference as sequence-specific guides in both transcriptional and posttranscriptional silencing mechanisms [26]. Several roles of AGO have been observed in translational regulation and RNA interference but their functions in human disease remain a top priority. Li, Yu, Gao and Li [23] studied the expression on AGO protein in colon cancer as a potential biomarker, Sun, et al. [27] reported the prognostic expression status of PIWIL1 in CRC and Völler, et al. [28] also studied their expression in cancer entitles. The information for the understanding of these processes is likely to improve as new structures of protein-nucleic acid complexes are solved and the structural details of the interactions are analyzed. However, experimental determination by high-resolution methods is a tedious and difficult process. 

Molecular simulation has emerged as an efficient and cost-effective tool in binding analysis from lead identification to optimization and beyond [29]. The process of molecular interaction through a non-covalent bond with high affinity and specificity to form a specific complex is crucial to all processes in living organisms [30]. Protein functions are majorly determined based on their binding interaction with other molecules or ligands [31]. Therefore, understanding protein-ligand interactions are central to understanding molecular biology. Additionally, information regarding the mechanisms of target interaction of protein-ligand binding is also likely to promote the discovery of drugs, a better understanding of gene silencing, the treatment and management efficacy in various diseases, most especially in cancer and the CRC subtype. This study, therefore, insights into an improved understanding at the molecular level the microRNA-assisted target recognition and regulation of argonaute as a therapeutic modality against CRC. Molecular docking approaches of microRNA conformations adopted within the binding pocket of the Argonaute protein could also assist to estimate the residual amino acids, hydrogen bond interactions and binding free energy to provide information crucial to the intermolecular recognition mechanism.

## 2. Results

### 2.1. Identification of Candidate MicroRNA and Target Genes

**Figure 1:**
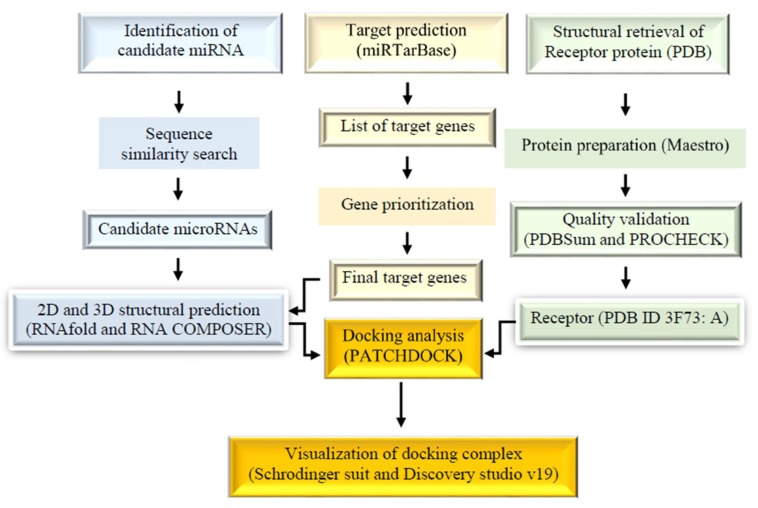
Schematic representation of the methodology.

**Figure 2:**
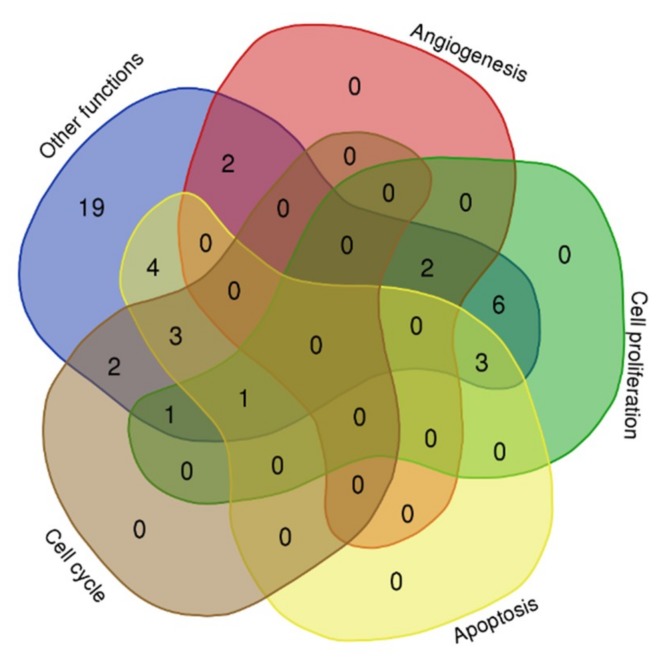
Biological processes of the microRNA target genes.

**Figure 3:**
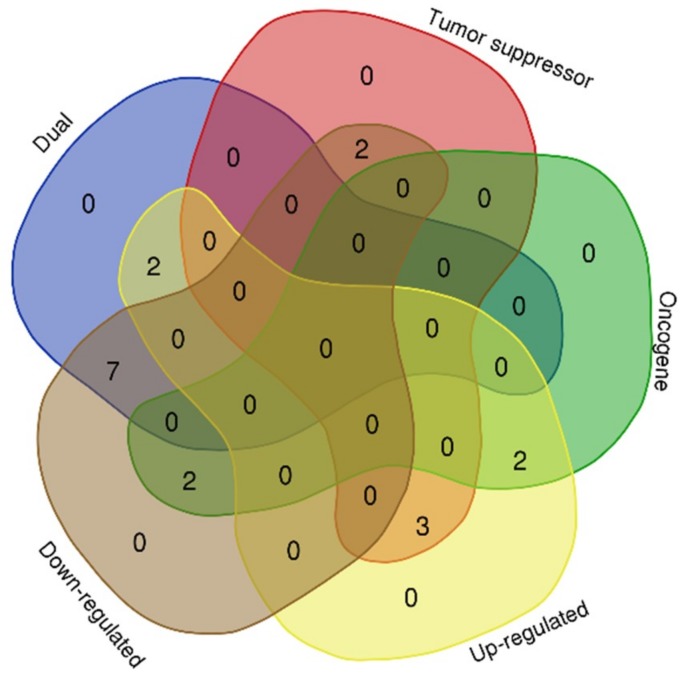
MicroRNA target genes involved in several cancer subtypes.

Figure 1 represents the overall methodology employed in this study. The sequence similarity search was employed through the basic local alignment search tool for nucleic acids (BLASTN) and the Homology Detection and Clustering Database at High Identity with Tolerance (CH-HIT-EST-2D) between the total microRNAs from miRBase as reference microRNAs and microRNAs experimentally validated in 4 databases (DbDEMC at http://www.picb.ac.cn/dbDEMC/, miR2Disease at http://www.mir2disease.org/, HMDD at http://www.cuilab.cn/hmdd, and miRCancer at http://mircancer.ecu.edu/) as the query set. With a similarity threshold of 0.90, the result was text-mined to obtain the final list of 5 candidate microRNAs together with their clusters associated with CRC (Table 1). MiRTarBase was used to predict the target genes of these microRNAs. Collectively, 44 genes alongside their minimum free energies (MFE) were identified to target candidate microRNAs after their intersection analysis with two CRC gene databases (CoReCG and gbCRC) (Table 2). The miR-1 targeted 12 genes, miR-2 targeted 10, miR-3 targeted 8, miR-4 targeted 6, and finally, miR-5 was associated with 8 genes. The combined targets were used as inputs in DAVID for the functional annotation as the first phase of gene prioritization. The result showed that 18 target genes were involved in cancer (GAD_DISEASE_CLASS) as shown in Table 3 with the *p*-value of 1.8E-3 and a Benjamini score of 1.6E-2. To further strengthen the involvement of the microRNAs in CRC and to further prioritize them for the candidate microRNAs, only genes that were enriched in CRC were considered. To finally select the genes of interest for the 5 microRNAs, the biological processes (Figure 2; Table 3), expression profile (Figure 3), MFE, and the number of experimentally validation methods were considered. The final list of microRNAs together with their target genes used for the docking analysis were shown in Table 4.

### 2.2. MicroRNA Target Genes Associated with CRC and Their MFE (miRTarBase)

The table above (Table 2) showed the target genes of the five microRNAs discovered through a sequence similarity search implicated in CRC. The miRTarBase prediction tool was used to verify the target genes. These target genes have been experimentally validated by one or more of the following validation methods: Reporter assay, western blot, qPCR, microarray, NGS and pSILAC. Each of the genes was also confirmed by their minimum free energy (MFE) in kcal/mol.

### 2.3. Biological Processes of the MicroRNA Target Genes

The target genes and their involvement in different biological process plotted using a Venn diagram (http://bioinformatics.psb.ugent.be/webtools/Venn/). The numbers denoted in the plot indicated the number of target genes involved in each of the different biological process (Figure 2).

### 2.4. Gene Enrichment in Cancer and Their Biological Functions

The involvement of 18 genes from the 44 target genes after gene prioritization through the DAVID database are presented in Table 3 alongside their involvement in different biological functions as reported from UniProt (http://www.uniprot.org/) web server (Table 3).

### 2.5. Expressions MicroRNA Target Genes Involved in Several Cancer Subtypes 

The expression profiles of the 18 genes (Table 3) were considered through an extensive literature search. The result of the search concluded that nine (FGF2, CHEK1, WT1, MDM2, BARD1, BMP2, CHEK4, BAMBI, and SOD2) of the genes have a dual role as oncogene and tumor suppressor genes. FGF2 [32,33] and CHEK4 [34] are up-regulated while the expression of CHEK1 [35,36], WT1 [37], MDM2 [38,39,40,41], BARD1 [42,43,44], BMP2 [45], BAMBI [46], and SOD2 [47,48,49], have been reported to be down-regulated in several cancer subtypes, including CRC. Furthermore, four (BUB1, RAN, REL, and RPS19) of the target genes were strictly oncogenic in nature showing that BUB1 [50,51] and RPS19 [52] were up-regulated and RAN [53], and REL [54] were down-regulated in solid tumors including CRC (Figure 3). The remaining five target genes are tumor suppressive in nature. This shows that XIAP [55] and KMT2A [56,57] were up-regulated while TP53 [58], HOXB13 [59,60], and VHL [61,62] were reportedly downregulated in cancers, including CRC. 

### 2.6. Binding Affinity and Structural Determination of MicroRNA and Duplex 

The binding energy (BE in Kcal/mol) and minimum folding energy (MFE in kcal/mol) of the microRNA target genes were exploited with two web-based tools namely, miRTarBase and RNAfold respectively. The secondary structures of the duplexes (microRNA-mRNA) were also revealed through the latter webserver (Table 5). The minimum folding energy of all the duplexes is high enough to be regarded as a good binding affinity between the candidate microRNAs and their targets. Therefore, the target genes have strong binding affinity for their respective microRNAs (miR-1 and HOXB13, miR-2 and mRNA, miR-3 and SOD2, miR-4 and BARD1, miR-5 and TP53). These duplexes were finally subjected to the molecular docking interaction.

### 2.7. Structural Model of MicroRNA-mRNA Duplexes

**Figure 4:**
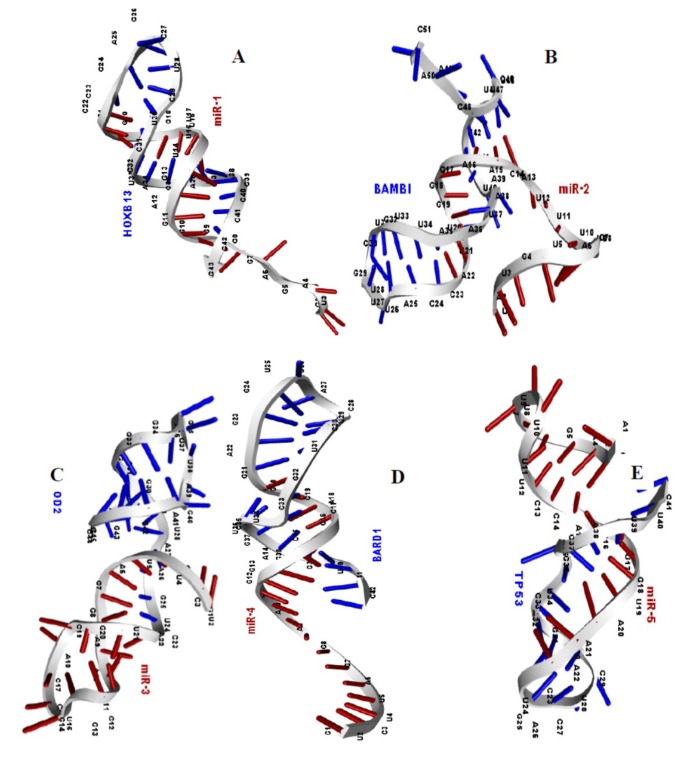
Structural model of miR-1 and mRNA of HOXB13 gene (**A**), miR-2 and mRNA of BAMBI gene (**B**), miR-3 and mRNA of SOD2 gene (**C**), miR-4 and mRNA of BARD1 gene (**D**), miR-5 and mRNA of TP53 gene (**E**), complexes are deciphered, respectively.

To reveal the three-dimensional structure of the microRNA-mRNA duplexes for the molecular ducking analysis, their binding sequences from the miRTarBase database were used as inputs in the RNAfold web server for a secondary folding pattern and dot-bracket. The secondary structure of the duplexes, their binding energy and the minimum folding energy in kcal/mol are reported in Table 5. The dot-bracket notation generated was also used as inputs in the prediction of the tertiary structure prediction [63] in RNA COMPOSER (Figure 4). The results of Table 5 (binding energies of the duplexes) and Figure 4 (the 3-D structure of the five microRNA-mRNA duplexes) suggested high binding affinity and strong molecular interaction between them.

### 2.8. Extraction and Preparation of AGO Protein Structure 

**Figure 5:**
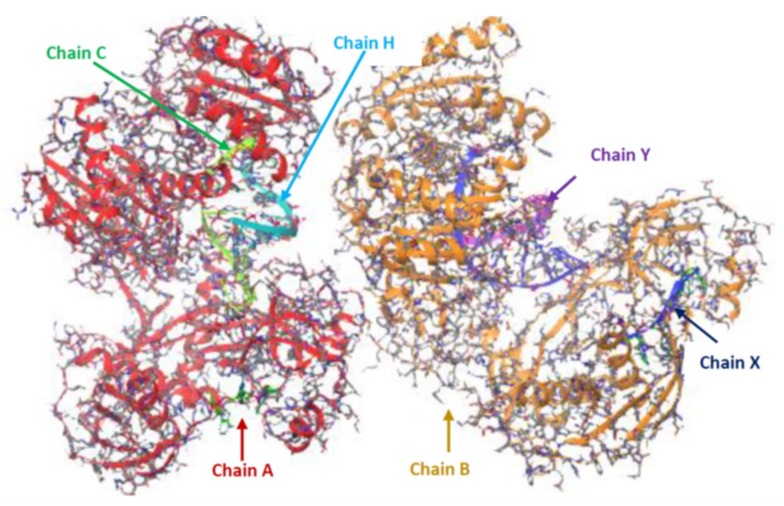
The receptor protein (3D-AGO protein) before preparation visualized by Maestro software.

**Figure 6:**
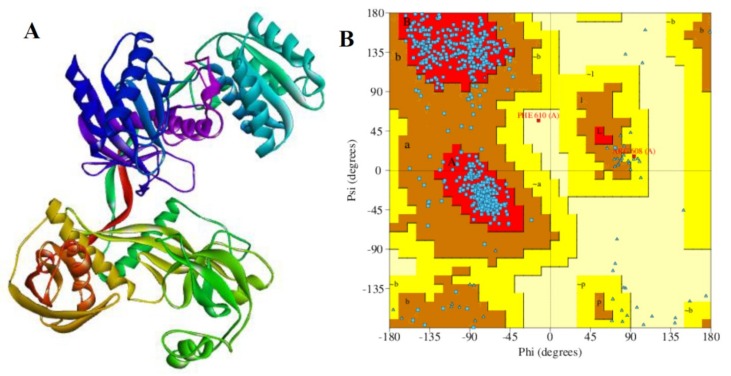
The structural details of the receptor. (**A**) Prepared chain A (Maestro, Discovery studio) and (**B**) its Ramachandran plot (PDBSum, PROCHECK). The quality of the prepared chain A was estimated by PDBSum server. The residues in most favored regions (A, B, L), the residues in additional allowed regions (a, b, l, p) and residues in generously allowed regions (~a, ~b ~l, ~p). The structural details of chain A (ID: 3F37: A) consist of 6 sheets, 9 gamma turns, 12 beta hairpins, 14 beta bulges, 33 strands, and 55 beta turns.

The 3D structure of the Argonaute protein was retrieved from the protein data bank (PDB ID: 3F73). In its raw state, AGO is a homodimer with two protein chains A and B, two nucleic acid groups, two molecules of co-factors Mg^2+^, a molecule of phosphate group and 16 water molecules (chain A, B, C, H, X, and Y). The structural preparation and necessary corrections were carried out using the Maestro Molecular Modelling tool (2019-2), a product of Schrödinger, and discovery Studio v19.1.0. The AGO protein files from PDB were not suitable for immediate use in the molecular modeling calculation due to the fact that they contain heavy atoms which include co-crystallized ligands, water molecule, metal ions, and co-factors. Further, the structure is a homo-dimer with missing atoms and connectivity information. Therefore, protein preparation wizard in Maestro, Schrodinger was used for the preparation and finally, it was reduced to a single chain (A). For the optimization of the H-bond network, PROPKA was employed to re-orientate hydroxyl and thiol groups, water molecules, amide groups of Asparagine and glutamine, and the imidazole ring of Histidine, as well as the prediction of the protonation states of histidine, aspartate, glutamate, and also the tautomeric states of histidine. The restrained minimization was finally performed to alleviate steric clashes and to relax side-chains (RMSD = 0.030 Å) and water molecules important to the binding receptor, was also maintained at 3. The AGO protein structure (raw and refined chain A) are depicted in Figure 5 and Figure 6 respectively.

### 2.9. Validation of Chain A of Argonaute Protein

The quality of the processed chain A was evaluated and validated using PROCHECK, a program that relies on Ramachandran plot for structure verification [64]. As shown in Figure 6A,B, the results from the PROCHECK ascertained that the prepared chain A has 91.5% residues in the most favored regions and 8.1% residues in the additional allowed regions. Further, 0.2% residues were found in the generously allowed regions and disallowed regions in each case. Therefore, the prepared protein is considered to be of high quality based on the percentage distribution of the amino acid residues. Furthermore, a G-factor that provides a measure of how unusual or conversely, how usual a given stereochemical property is [65], was also determined using this program. A G-factor of less than −0.5 is unusual and less than −1.0 indicates highly unusual. However, the generate G-factor for the prepared chain A of the receptor protein was −0.34 for dihedral angels, −0.04 for main chain covalent forces and −0.20 overall.

### 2.10. Docking Analysis Between Receptor Protein and MicroRNA

**Figure 7:**
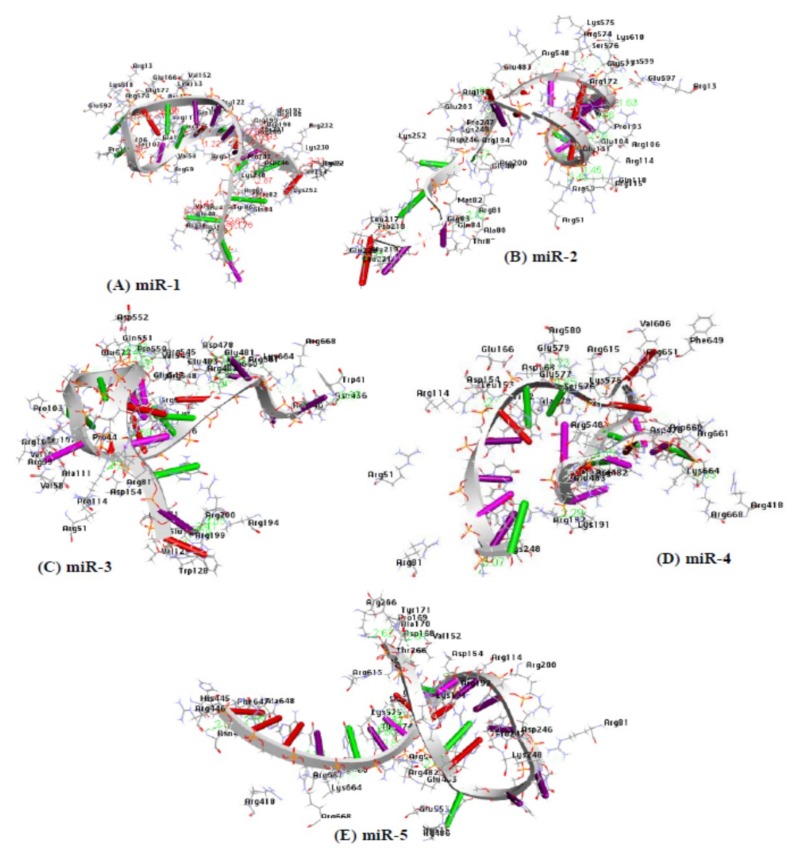
The amino acid residues of Argonaute protein participating in the interaction with each of the five candidate microRNAs within a distance of 3.5 Å are deciphered, respectively. (**A**) amino acids participating in miR-1-Agonaute protein duplex, (**B**) amino acids participating in mir-2-Agonaute protein duplex, (**C**) amino acids participating in mir-3-Agonaute protein duplex, (**D**) amino acids participating in mir-4-Agonaute protein duplex, and (**E**) amino acids participating in mir-5-Agonaute protein duplex.

PatchDock as molecular docking method was used for the docking interaction between the microRNAs and the AGO protein. The PDB file of the AGO protein and each of the candidate microRNAs was used as inputs. The root mean square deviation (RMSD) is often used to measure the quality of reproduction of the correct pose and to validate the docking protocol. For a true binding pose to be good, the RMSD must be low, therefore, the clustering RMSD was adjusted to 1.5 Å. The method of PatchDock relies on the shape complementarity theory [66]. A previous study also reported the reliability and usability of the Patch dock tool in molecular docking analysis [67]. The result files generated for each of the microRNAs were ranked according to their geometric shape complementarity score. For the first round of docking, the result with the highest score (geometric shape complementary) was chosen as the best microRNA-AGO complex [67] for each of the five candidate microRNAs (Table 6). The strong binding affinity of these results was observed through their scores and the amino acid residues involved in the interaction between the microRNAs and the AGO protein. As evident, the presence of strong hydrophobic amino acids (mir-1: 21; mir-2: 20; mir-3: 27; mir-4: 22; and mir-5: 27) and amino acids with aromatic side chains (miR-1: 7; mir-2: 3; mir-3: 6; mir-4: 4; and mir-5: 7) within the distance of 3.5 Å (Figure 7; Table 7), and the hydrogen bond within the distance of 2.0 Å are supportive that gene regulation through the argonaute protein are driving by microRNA (Table 8).

### 2.11. Hydrogen Bond Interaction

**Figure 8:**
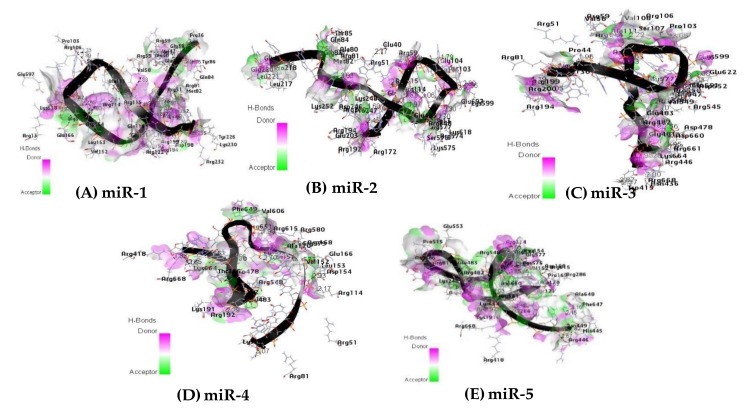
Hydrogen bond interaction between the residual amino acids of the receptor protein and the microRNAs (**A**) miR-1, (**B**) miR-2, (**C**) miR-3, (**D**) miR-4, and (**E**) miR-5 respectively (3.5 Å distance).

**Figure 9:**
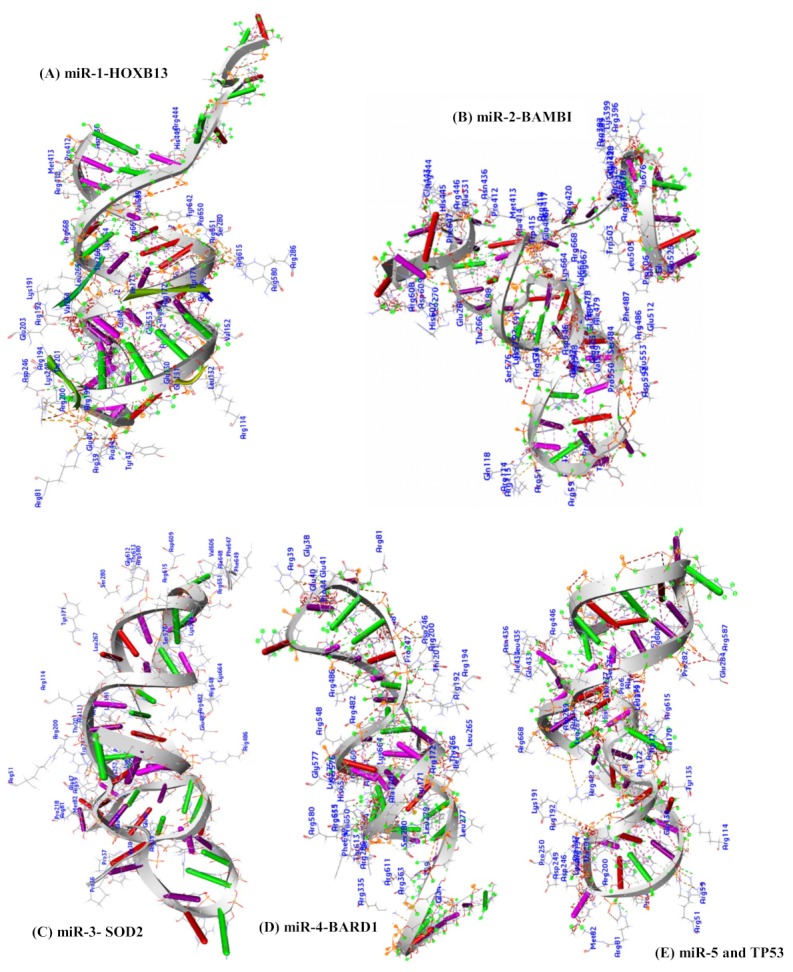
Docking complex results of microRNA-mRNA and the receptor (Chain A of argonaute protein). The amino acid residues participating in the interaction between the receptor and miR-1-HOXB13 (**A**); Residual amino acids participating in the interaction between the receptor and miR-2-BAMBI (**B**); miR-3-SOD2 (**C**); miR-4-BARD1(**D**); and miR-5- TP53 (**E**).

Hydrogen-bonds (H-bond) are an important interaction which dictate the specificity of ligand binding. Their important contribution is explicitly incorporated into the molecular simulation to enhance the binding of molecules to their receptors in an energetically favorable manner [68]. For protein-ligand interactions, hydrogen bonds have been thought to play some significant roles. These roles include the orientation of the binding molecule, ligand recognition, and binding affinity. The latter is one of the most important issues to be considered in protein-ligand interaction. The highest number of hydrogen bond interactions were found among the interacting atoms of miR-1, miR-3 and the residual amino acid of the receptor protein binding pocket with 45 H-bond and 35 H-bond respectively (Figure 8A,C). For miR-2, miR-5 and receptor protein, a total of 28 H-bonds were involved (Figure 9B,E), while the lowest number of hydrogen bonds (18 H-bonds) was observed among the interacting atoms of miR-4 and the receptor protein (Figure 8D). All the hydrogen bonds observed in Figure 8 are within the distance of 3.5 Å. Table 7 shows the residues of the amino acids involved in hydrogen bonding between the Argonaute protein and the microRNAs within the distance of ≤ 2.0 Å. The hydrogen bonds are key to the determination of the interaction (protein-ligand) therefore, they are fundamental to the biological process [69]. The results revealed that the higher the number of favorable interactions, the more the hydrogen bonds. This result may, therefore, support the mechanism by with microRNAs regulates gene expression through RISC.

### 2.12. Docking Analysis between Argonaute Protein and MicroRNA-mRNA Complex

Similar to the docking analysis of microRNAs to the argonaute protein, the microRNA-mRNAs complexes between the candidate microRNAs and their target genes were further docked against the argonaute protein and possible binding interaction in terms of hydrophobicity, aromatic residual amino acids, and hydrogen bonding was analyzed. The docking was carried out on the argonaute protein (chain A) and miR-1-HOXB13; miR-2-BAMBI; miR-3-SOD2; miR-4-BARD1; and miR-5-TP53 separately in PATCHDOCK. Based on the geometric scoring analysis, the highest score for each of the complexes were reported in Table 9.

In nature, strong hydrophobic amino acids together with amino acids with aromatic side chains are important to binding interactions in terms of stability between the receptor and the ligand. Therefore, the binding interaction between the 5 complexes (miR-1-HOXB13, miR-2-BAMBI, miR-3-SOD2, miR-4-BARD1, and miR-5-TP53) and argonaute protein (chain A) was investigated by examining the residual amino acids in the binding pocket of the argonaute protein within the distance of 3.5 Å. The residual strong amino acids of the receptor (argonaute protein) VAL42, LEU45, VAL129, LEU132, ALA133, VAL147, ALA151, VAL152, LEU153, TRP156, ALA170, TYR171, ILE173, LEU174, VAL193, VAL264, LEU265, LEU267, LEU277, LEU279, LEU281, ALA331, ALA414, ILE434, ALA479, VAL549, VAL573, VAL606, LEU617, ALA644, LEU652, LEU658, VAL663, and VAL685 in miR-1 and HOXB13 complex; VAL42, LEU45, LEU46, ALA47, ALA50, VAL58, ALA111, LEU132, LEU189, LEU204, LEU205, VAL264 LEU265, LEU267, LEU270, LEU277, ALA331, LEU389, LEU395, ALA414, LEU421, ALA423, LEU424, LEU435 LEU439, ALA479, LEU505, ALA508, LEU522, VAL549, ALA648, ALA659, LEU662, VAL663, VAL666, ILE671, LEU674, and VAL677 in miR-2 and BAMBI complex; VAL42, LEU45, LEU46, ALA47, VAL58, VAL108, ALA111, LEU112, LEU132, ALA133, VAL152, ALA170, ILE173, LEU215, LEU217, ILE254, LEU265, LEU267, LEU279, LEU281, VAL606, LEU617, ALA648, and LEU652 in miR-3 and SOD2 complex; VAL42, LEU45, VAL147, VAL152, ALA170, ILE173, VAL264, LEU265, LEU267, LEU277, ALA278, LEU279, LEU281, ILE434, LEU435, ALA450, LEU452, VAL573, VAL606, ALA644, ALA648, LEU652, and VAL685 in miR-4 and BARD1 complex; VAL42, LEU45, VAL147, VAL152, ALA170, ILE173, VAL264, LEU265, LEU267, LEU277, ALA278, LEU279, LEU281, ILE434, LEU435, ALA450, LEU452, VAL573, VAL606, ALA644, ALA648, LEU652, and VAL685 in miR-5 and TP53 complex (3.5 Å). Similarly, amino acids such as TYR43, TYR135, TRP202, TRP415, TYR642, PHE647, PHE649, and PHE684 in miR-1 and HOXB13 complex; TYR43, TRP182, TRP202, TRP415, TRP447, PHE485 PHE487, TRP503, PHE610, PHE647, PHE649, and PHE683 in miR-2 and BAMBI complex; TYR43, TYR86, TYR171, TRP202, TRP243, TRP415, PHE487, PHE647, and PHE649 in miR-3 and SOD2 complex; TYR43, TYR135, TYR171, TRP202, PHE360, TRP447, PHE487, PHE610, TYR642, and PHE649 in miR-4 and BARD1 complex; and TYR43, TYR135, TYR171, TRP202, TRP283, TRP447, PHE610, TYR642, PHE647, and PHE649 in miR-5 and TP53 with aromatic ring are also found as participating in the interaction within the binding pocket of the receptor protein (3.5 Å) (Table 8 and Figure 9).

## 3. Discussion

The study aimed to predict the mechanism of gene regulation mediated through microRNAs involved in CRC using the in silico approach. Since the discovery of microRNA, several studies have reported their involvement in a variety of physiological and pathological processes and mutations affecting their normal expression which may be critical to their role in the development of human diseases [70,71,72], such as cardiovascular diseases [73,74], neurodegenerative diseases [75,76,77], and several cancer subtypes [21,72,78,79]. Additionally, many studies have investigated the diagnostic [80,81] and therapeutic roles [82] of this non-coding RNAs in human diseases. These microRNAs are able to control gene expression in a sequence-specific manner, most especially in the mechanism of gene silencing by forming RISC comprising the argonaute protein [83]. Experimental approaches have been used to study the RNA induced silencing complex at both the molecular and atomic levels [84,85,86].

In light of this, the molecular interaction between chain A of the argonaute protein, microRNAs and the target genes were investigated in CRC with the in silico approach.

In this study, the results of BLASTN and CD-HIT-EST-2D were obtained from 125 validated query sequences and 2226 total microRNA sequences as the reference microRNAs. The microRNAs obtained from BLASTN were based on the parameters: (1) The expected value of 1e-2, (2) the word size of 7 and (3) a similarity index between 90%–99%. The result of the CD-HIT-EST-2D obtained was based on a threshold of 0.90 and word size of 7.

Five microRNAs (Table 1) were finally retained after a sequence similarity search based on their uniqueness after text-mining to show their non-involvement in CRC. These microRNAs targeted 44 genes (Table 2) which were further reduced to five based on the set criteria (involvement in CRC, expression pattern, MFE, biological processes, and their validation methods). The minimum free energy (kcal/mol) of the binding affinity of both the target genes and their microRNAs were further studied in other to verify their interaction strength. After studying the biological processes of these genes, their enrichment in cancer and CRC were also identified through the DAVID database.

The aberrant expression of HOXB13 is associated with CRC [87]. The expression of this gene in the early embryonic development of the intestine represents embryogenic phases in an important tissue-specific marker [88]. Therefore, it strongly correlates with lymph nodes metastasis (TNM) [89]. The over-expression of BAMBI has also been detected in colorectal cancer [90]. This gene has further been linked with late-stage (M) in CRC [91,92]. The expression of SOD2 is increased in pre-malignant (T and N) stages during colorectal carcinogenesis whereas SOD1 is expressed only in colorectal tumors [93]. The aberrant expression of BARD1 is associated with the drivers of various types of cancer [94]. TP53 is correlated with overall survival in stage II and III CRC patients [95].

The selected microRNA target genes were involved (HOXB13, BAMBI, SOD2, BARD1, and TP53) in various biological processes which are crucial to carcinogenesis in CRC. The minimum free energies of −23.9 kcal/mol for HOXB, −13, 9.6 kcal/mol for BAMBI, −16.2 kcal/mol for SOD2, −21.3 kcal/mol for BARD1, and −10.7 kcal/mol for TP53 confirmed that that the binding interaction between the candidate microRNAs and their target genes were energetically favorable, which can be confirmed by the binding energies of each duplex (Table 5). Additionally, to investigate the mechanism by which the candidate microRNAs miR-1, miR-2, miR-3, miR-4, and miR-5 bind argonaute protein in RNA induced silencing complex to target specific genes namely, HOXB13, BAMBI, SOD2, BARD1, and TP53, their molecular interactions were studied. 

Prior to the molecular study, microRNAs and microRNA-mRNA duplexes were converted to PDB format. The argonaute protein (receptor) was also downloaded alongside from the protein data bank. As the raw structure is a homodimer consisting of heavy atoms (co-factors, water molecules, metal ions, and co-crystallized ligands) and is of limited resolution due to the x-ray crystallography experiment, the structure was checked (Figure 5). 

Specifically, the protein preparation wizard in MAESTRO was used to optimize the hydrogen bond network (PropKa), and alleviate the steric clashes (restrained minimization) by force field: OPLS_2005, Epik was used to generate the het states and finally, missing atoms were fixed using PRIME. The prepared protein was validated using PROCHECK and PDBSum (Figure 6).

The docking algorithm (PATCHDOCK) was employed to computationally study the miRNA-protein and microRNA-mRNA-protein interactions. In order to estimate the strength of the interactions between the receptor and microRNAs, the molecular docking results (argonaute and microRNA and microRNA-mRNA) were estimated by examining the structural components and binding affinity [96].

The general interactions between the receptors and ligand include hydrophobic, hydrogen, pi stacking, weak hydrogen bond, salt bridge, amide stacking, and cation pi. The molecular docking results of the receptor-microRNA interaction and receptor-(microRNA-mRNA) interaction indicated that the non-covalent interactions include hydrophobic interactions between the residual amino acids of the protein and specific atoms of the microRNA and or mRNAs. The hydrogen bonds and the pi stacking bonds, which are the most common interactions, are also observed in the binding analysis to prove that microRNA is crucial to gene regulation.

Rath et al. [97] reported that the presence of aliphatic amino acids such as, isoleucine, leucine, valine, and alanine, which are strong hydrophobic in nature, confer stability during molecular interaction in protein-ligand binding. Further, amino acid residues which are relatively hydrophobic with aromatic side chains such as tryptophan, tyrosine, and phenylalanine provide steadiness towards the binding stability within the binding pocket of a protein.

From the docking analysis of the receptor and the candidate microRNAs, the presence of strong and relatively strong hydrophobic residual amino acid and aromatic rings observed between the candidate microRNAs and the receptor protein (chain A of argonaute protein) together with hydrogen bond interactions within the distance of 3.5Å (Table 7, Figure 7) are proofs that the molecular interaction involved are favorable and stable at the atomic level respectively. 

The amino acid residues of the receptor participating in the hydrogen bonding interaction with the candidate microRNAs at the molecular level within the distance of 2.0 Å are also reported in Table 8. This H-bonding interaction strongly assists in receptor stability through the candidate microRNAs during gene regulation (Figure 8). Previous studies have reported that hydrogen bonding between the interaction of two molecules, such as protein and ligand, are important interactions driving potent binding and selectivity [98] and stabilizing ligand conformation [99]. Furthermore, the presence of strong hydrophobic amino acids namely; LEU 45, LEU 265, and LEU 267 and aromatic rings of amino acids TYR 43, TRP 202 and PHE 649 (relatively strong hydrophobic) during molecular interaction of the receptor with miR-1 and HOXB13, miR-2 and BAMBI, miR-3 and SOD2 miR-4 and BARD1, and miR-5- TP53 complex (Table 9, Figure 9 and Table 10) are observed to be commonly participating in all the microRNAs together with their targets in the receptor-binding pocket within the distance of 3.5Å. The hydrophobic contacts are the most common interactions in protein-ligand complexes. The most common hydrophobic interaction is the one formed by an aliphatic carbon in the receptor and an aromatic carbon in the ligand. Leucine, followed by valine, isoleucine and alanine side-chains are the most frequently engaged in hydrophobic interactions [100]. 

## 4. Materials and Methods

### 4.1. MicroRNA Identification

A sequence similarity search was employed to identify candidate microRNAs between the total microRNA sequences (obtained from miRBase) at http://www.mirbase.org/ and microRNAs associated with CRC obtained from four different experimentally validated CRC microRNA databases (dbDEMC, HMDD, miR2Disease, and miRCancer). The parameters for the command line include a sequence identity threshold of 0.90; an E-value of 1e-3; a word size of 7. 

### 4.2. Target Prediction and Correlation to CRC

The miRTarBase is an experimentally validated microRNA-target interactions database at http://mirtarbase.mbc.nctu.edu.tw/php/index.php. Generally, this database is composed of targets experimentally validated through reporter assay, western blot, microarray, and next-generation sequencing experiments [101,102]. The gene browser for CRC (gbCRC) at http://gbcrc.bioinfo-minzhao.org/ and CRC for the gene database (CoReCG) at http://lms.snu.edu.in/corecg/gene are databases containing only validated CRC genes. 

The miRTarBase, gbCRC, and CoReCG were used to identify and correlate the targets of the microRNAs.

Furthermore, the targets prioritization was carried out using the Database for Annotation, Visualization and Integrated Discovery (DAVID) at https://david.ncifcrf.gov/ for functional enrichment in CRC [103,104]. To finally select the genes of interest, the expression profile, the biological processes, the minimum free energy score based binding affinity between the targets and the microRNAs (MFE), and the number of experimentally validation methods were considered. 

An intersection analysis tool accessed at http://bioinformatics.psb.ugent.be/webtools/Venn/ was used to create Venn diagrams of the involvements of the target genes in the cancer subtypes and their functions.

### 4.3. Structural Prediction of Candidate MicroRNA and Target Complexes

To determine the secondary structure and the dot-bracket notation of both the microRNAs and their targets, the RNAfold web server was employed. This software at http://rna.tbi.univie.ac.at//cgi-bin/RNAWebSuite/RNAfold.cgi is used to predict the secondary structure of single-stranded RNA or DNA sequences, including their folding energy. The dot-bracket annotations generated were therefore used as inputs in the RNA-COMPOSER (http://rnacomposer.cs.put.poznan.pl/) to generate the 3-dimensional structures of their duplex. 

### 4.4. Protein Selection and Preparation

The sole component of the RNA induced silencing complex (RISC) (AGO protein) was retrieved in PDB format from the protein data bank with the ID: 3F73 (DOI: 10.2210/pdb3F73/pdb) at https://www.rcsb.org/structure/3f73. The molecule was further prepared and visualized using Schrödinger, 2019 suit and discovery studio v19. The protein preparation wizard in Maestro was to optimize the hydrogen bond network (PropKa), alleviation of steric clashes (restrained minimization) by force field: OPLS_2005, Epik was used to generate the het states and finally, missing atoms were fixed using PRIME. The prepared protein was validated using PROCHECK and PDB Sum.

### 4.5. Molecular Docking 

*In silico* protein-ligand docking was performed using the webserver PATCHDOCK at https://bioinfo3d.cs.tau.ac.il/PatchDock/php.php. The molecular docking between the receptor protein (chain A of the AGO protein) and the microRNA and the microRNA-mRNA complex with chain A of the AGO protein was carried out. The PATCHDOCK software is based on the shape complementarity of the interactions to generate the best candidate solution [67]. The clustering root-mean-square deviation (RMSD) was chosen to be 2.0 Å with the complex type protein-small ligand. The microRNAs, microRNA-mRNA, and AGO were all converted to PDB file formate and were used as inputs into the PATCHDOCK webserver. The results generated were presented in PDB based on the geometric shape complementary score, the approximate interface area (AI area), and the atomic contact energy with their transformation files. The pose with the highest score was considered as the best complex [67]. 

Finally, the interactions (including receptor surface (hydrogen bond and charge) and binding observed in the docked conformations in the PDB format were analyzed and inspected with Maestro and PDB sun and visualized using the discovery studio v19 software.

### 4.6. Statistical Analysis

For microRNA selection, the BlastN parameters were set at 1e-2 for expected value, 7.0 for word size, and 90–99% for similarity index. The CD-HIT-EST-2D parameters were set at 0.90 for threshold and 7.0 for word size. The genes considered in DAVID were regarded statistically significant at *p*-value of 1.8E-3 with the Benjamini score of 1.6E-2. The root-mean-square deviation (RMSD) was set at 0.030 Å for the protein preparation in Schrödinger (restrained minimization). The protein quality check at PROCHECK was also considered significant at 90% and above for residues in the most favored regions. In PatchDock, RMSD was adjusted to 1.5 Å. In discovery studio, the amino acid residues were considered within the distance of 3.5 Å, while hydrogen bonding was considered between 2–3.5 Å. A value of *p* < 0.05 was considered to indicate a statistically significant difference.

## 5. Conclusions

The study identified 5 microRNAs involved in CRC along with 5 target genes prioritized with some set criteria. The molecular docking analysis confirmed that these microRNAs could assist the RNA induced silencing complex (Argonaute protein as the sole) in targeting these genes for regulation. This was confirmed by the predominant hydrophobic interaction within the receptor pocket which made a substantial contribution in stability with microRNA-mRNA duplexes while hydrogen bonding and polar interactions assisted in the proper orientation of the binding interaction. These interactions at the molecular level are important in protein folding and structural stability and also in mediating the binding of the protein to their targets. This result may further serve as a lead to the experimental approach in understanding the molecular mechanism of action of gene regulation in CRC.

## Figures and Tables

**Table 1:** MicroRNAs and their clusters.

Candidate miRNA	Validated microRNA	Fasta Sequences
miR-1	hsa-miR-193a-5p	>hsa-miR-193a-5p MIMAT0004614 UGGGUCUUUGCGGGCGAGAUGA
miR-2	hsa-miR-450b-3p	>hsa-miR-450b-3p MIMAT0004910 UUGGGAUCAUUUUGCAUCCAUA
miR-3	hsa-miR-501-3p	>hsa-miR-501-3p MIMAT0004774 AAUGCACCCGGGCAAGGAUUCU
miR-4	hsa-miR-501-3p	>hsa-miR-501-3p MIMAT0004774 AAUGCACCCGGGCAAGGAUUCU
miR-5	hsa-miR-513a-3p	>hsa-miR-513a-3p MIMAT0004777 UAAAUUUCACCUUUCUGAGAAGG

**Table 2:** MicroRNA target genes associated with colorectal cancer (CRC) and their MFE (miRTarBase).

miR-1	MFE	miR-2	MFE	miR-3	MFE	miR-4	MFE	miR-5	MFE
A1CF	−19.10	BAMBI	−9.80	SOD2	−16.20	BARD1	−21.30	PDCD4	−11.80
PAQR3	−13.80	XIAP	−8.70	PAQR3	−10.80	SLC1A5	−17.30	VMP1	−12.50
STMN1	−19.90	BMP2	−8.70	SLC7A11	−20.60	WT1	−14.80	CDK4	−10.70
MACC1	−18.00	ZNF703	−13.80	MDM2	−11.90	CLMN	−16.40	TP53	−10.70
FGB	−12.90	PPM1D	−16.90	RAN	−14.70	REL	−19.80	CHEK1	−8.70
HOXB13	−23.90	BUB1	−8.00	LAMB1	−11.52	HDGF	−21.70	H2AFZ	−9.60
ALDOA	−19.20	LYN	−12.90	ORAI2	−19.50			RNF138	−18.20
CHAC1	−20.10	KLF8	−11.02	VAV3	−17.80			SLC7A5	−12.50
GSTK1	−18.10	FGF2	−14.60						
RPS19	−19.10	KMT2A	−17.02						
CRKL	−15.40								
VHL	−19.90								

MFE score based binding affinity between 5 miRNAs and 44 target genes associated with CRC as indicated by miRTarBase.

**Table 3:** Gene enrichment in cancer and their biological functions.

Gene	Function	miRNA	MFE
TP53	Cell cycle, Apoptosis, Cell proliferation, others	miR-5	−10.70
FGF2	Angiogenesis, Cell proliferation, others	miR-2	−14.60
CHEK1	Cell cycle, Apoptosis, other functions	miR-5	−8.70
WT1	Apoptosis, Cell proliferation, others	miR-4	−14.80
MDM2	Cell cycle, Cell proliferation, others	miR-3	−11.90
BARD1	Cell cycle, Apoptosis, others	miR-4	−21.30
BUB1	Cell cycle, others	miR-2	−8.00
XIAP	Apoptosis, others	miR-2	−8.70
BMP2	Cell proliferation, others	miR-2	−8.70
CDK4	Cell cycle, others	miR-5	−10.70
HOXB13	Angiogenesis, others	miR-1	−23.90
KMT2A	Apoptosis, others	miR-2	−17.02
VHL	Angiogenesis, others	miR-1	−19.90
BAMBI	Other functions	miR-2	−9.80
RAN	Other functions	miR-3	−14.70
REL	Other functions	miR-4	−19.80
RPS19	Other functions	miR-1	−19.10
SOD2	Other functions	miR-3	−16.20

**Table 4:** The final list of microRNAs and target genes.

S/N	miRNAs	Target Gene
1	miR-1	HOXB13
2	miR-2	BAMBI
3	miR-3	SOD2
4	miR-4	BARD1
5	miR-5	TP53

**Table 5:** Study of binding affinity between miRNA-mRNA duplex.

Gene	miRNA	Dot-Bracket Notation	2° Structure of Duplex	BE	MFE
HOXB13	miR-1	.......((((..((((((((........))))))))..))))	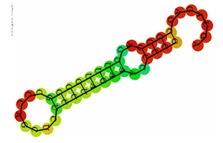	−13.3	−23.9
BAMBI	miR-2	............(((((.((((.((.....)).))))..))))).......	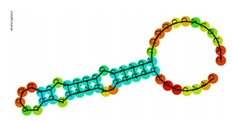	−2.3	−9.6
SOD2	miR-3	...((((..(((......))).)))).((.(((......))).))...	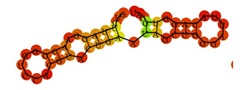	−8.5	−16.2
BARD1	miR-4	.............((((((((((....)))))...)))))..	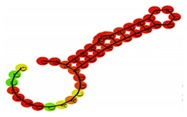	−12.8	−21.3
TP53	miR-5	.(((((...)))))..(((...(((...)))....)))...	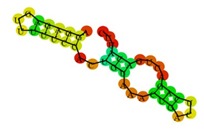	−4.0	−10.7

Note: BE- Minimum binding energy in kcal/mol; MFE- Minimum free energy in kcal/mol.

**Table 6:** The docking scores between miRNA and AGO protein.

miRNA-mRNA and AGO	Score	Area	ACE
miR-1 -AGO	19544	3390.80	−258.22
miR-2-AGO	18618	2832.70	−22.43
miR-3-AGO	18420	2814.10	−151.43
miR-4-AGO	18024	2344.20	−131.18
miR-5-AGO	20.372	2913.20	−488.07

The score indicates the geometric shape complementary score and atomic contact energy (ACE) score generated for each miRNA and AGO complex. miRNA, microRNA; AGO, argonaute; ACE, atomic contact energy.

**Table 7:** Molecular docking analysis results of microRNA and receptors with participating aa residues (3.5 Å).

miRNA	Hydrophobic AA	Aromatic AA	H-Bond
	(21 ^a^), LEU45 ^d^, ALA47 ^d^, VAL58 ^d^, VAL108 ^d^, ALA111 ^d^, LEU112 ^d^, VAL129 ^d^, LEU132 ^e^, ALA133 ^e^, LEU217 ^d^, ALA245 ^d^, ILE254 ^d^, VAL264 ^d^, LEU596 ^d^	(7b), TYR43 ^d^, TYR135 ^d^, TRP156 ^e^, TRP202 ^d^	(25 ^c^) ARG114 ^d^, ARG574 ^d^, GLY577 ^d^, LYS248 ^d^, ASP246 ^d^, ASP154 ^d^, ARG200 ^d^, GLY131 ^d^, PRO103 ^d^, LEU153 ^d^
**miR-2**	(20 ^a^), ALA47 ^d^, VAL58 ^d^, VAL108 ^d^, ALA111 ^d^, LEU112 ^d^, LEU132 ^e^, ALA133 ^e^, VAL152 ^d^, LEU153 ^d^, LEU217 ^d^, ALA245 ^d^, ILE254 ^d^, VAL264 ^d^, VAL549d ^d^, LEU596 ^d^, VAL620 ^d^	(3 ^b^), TYR43 ^d^, TRP156 ^e^, TRP202 ^d^	(21 ^c^) ARG114 ^d^, ARG574 ^d^, GLY577 ^d^, LYS248 ^d^, ASP246 ^d^, ARG548 ^d^, GLU483 ^d^, SER576 ^d^, ARG192 ^d^, LYS599 ^d^, ARG81 ^d^
**miR-3**	(27 ^a^), ALA47 ^d^, VAL58 ^d^, LEU64 ^d^, VAL108 ^d^, ALA111 ^d^, LEU112 ^d^, VAL129 ^d^, LEU132 ^e^, ALA133 ^e^, VAL152 ^d^, LEU153 ^d^, ALA450 ^d^, ALA479 ^d^, VAL549 ^d^, VAL620 ^d^, LEU652 ^d^, VAL663 ^d^	(6 ^b^), TYR43 ^d^, TRP156 ^e^, TRP447 ^d^	(26 ^c^) ARG114, ARG574, GLY577, ASP154, ARG548 GLU483, LYS664 ARG661, ARG200 GLY131 PRO103, LYS599 ARG81, ASP660,
**miR-4**	(22 ^a^), ALA47 ^d^, LEU132 ^e^, ALA133 ^e^, ALA151 ^d^, VAL152 ^d^, LEU153 ^d^, ALA170 ^d^, ILE173 ^d^, VAL264 ^d^, LEU265 ^d^, LEU267 ^d^, LEU279 ^d^, ALA479 ^d^, VAL573 ^d^, ALA648 ^d^, LEU652 ^d^, LEU662 ^d^, VAL663 ^d^	(4 ^b^), TYR135 ^d^, TRP156e, PHE649 ^d^	(15 ^c^) ARG114, LYS248, ARG548 GLU483, SER576 ARG192, LYS664 ARG661, LEU153, THR266 LYS575 ARG482
**miR-5**	(27 ^a^), LEU132 ^e^, ALA133 ^e^, ALA151 ^d^, VAL152d, LEU153 ^d^, ALA170 ^d^, ILE173 ^d^, VAL264 ^d^, LEU265 ^d^, LEU267 ^d^, LEU279 ^d^, ALA450 ^d^, ALA479 ^d^, VAL549 ^d^, VAL573 ^d^, ALA648 ^d^, LEU652 ^d^, LEU662 ^d^, VAL663 ^d^	(7 ^b^) TYR135 ^d^, TRP156 ^e^, TRP447 ^d^, PHE649 ^d^	(17 ^c^) ARG574 ^d^, ASP246 ^d^, ASP154 ^d^, SER576 ^d^, ARG192 ^d^, LYS664 ^d^, ARG661 ^d^, ASP660 ^d^, THR266 ^d^, LYS575 ^d^, ARG482 ^d^

^a^ Total number of residual hydrophobic amino acids involved in the interaction between the receptor and the candidate microRNAs; ^b^ Total number of aromatic amino acids involved in the interaction between the receptor and the candidate microRNAs; ^c^ Total number of hydrogen bond observed in the interaction between the receptor and the candidate microRNAs; ^d^ The residual amino acids of the receptor protein common to more than one interaction between microRNA binding to receptor; ^e^ The residual amino acids of the receptor protein common to all the microRNA binding to AGO.

**Table 8:** Hydrogen bond interaction between the amino acid residues of the receptor and the candidate microRNAs within the distance of 2.0 Å.

microRNA	AA Residues	Atoms	Distance	NA Residues
**miR-1**	GLN84	HE21-OP1	1.8	(G3)
	ARG574	HH11-O3’	1.7	(G12)
	ALA111	HA-O2’	1.9	(G16)
	PRO36	O-H4’	1.8	(G2)
	ASP154	OD1-H5’	2.0	(A11)
		O-H4′	2.0	(G2)
**miR-2**	GLY104	HA3-O6	1.8	(G15)
	ARG114	HD3-O4’	1.6	(G19)
	ARG574	HD3-OP1	1.5	(A10)
	GLU483	OE1-H5	2.0	(A8)
	ARG59	O-H4’	1.9	(A17)
**miR-3**	ARG548	HH11-O2’	1.9	(A17)
	ARG574	HH22-O4’	1.9	(G)
	VAL129	O-HO5’	1.8	(A1)
	ASP154	OD1-HO2’	1.6	(A13)
	PRO44	HA-O3’	2.0	(C4)
	GLY577	HA2-O2’	2.0	(A14)
	ARG661	HA-O2’	1.9	(U19)
	GLU622	OE1-H5’	1.8	(G9)
	ASP660	O-H2’	2.0	(U19)
**miR-4**	ARG668	HH12-O5’	1.8	(G1)
	ARG615	HD2-OP2	2.0	(A12)
	THR266	OG1-H5’	2.0	(G8)
**miR-5**	LYS575	HZ1-O2	1.9	(U6)
	ARG661	HE-O4’	1.9	(U4)
	ARG574	HD2-O4’	2.0	(A8)
	SER576	H-O2	2.0	(C7)

**Table 9:** Docking scores between miRNA-mRNA and AGO protein.

miRNA-mRNA and AGO	Score	Area	ACE
miR-1-HOXB13-AGO	24046	3962.90	−851.20
miR-2-BAMBI-AGO	24380	5528.70	−966.63
miR-3-SOD2-AGO	27570	3974.80	−652.52
miR-4-BARD1–AGO	24816	3524.00	−836.85
miR-5-TP53-AGO	23716	3402.30	−547.97

Score indicates the geometric shape complementary score and ACE score generated for each miRNA-mRNA and AGO complex. miRNA, microRNA; AGO, argonaute; ACE, atomic contact energy.

**Table 10:** Amino acid residues of the binding pocket of the argonaute protein involved in the molecular interaction with the microRNA-mRNA complex (3.5 Å).

miRNA-mRNA	Residual Hydrophobic AA	Aromatic AA
**miR1-HOXB13**	(34 ^a^), VAL42 ^c^, LEU45 ^d^, LEU132 ^c^, ALA133 ^c^, VAL147 ^c^, ALA151 ^c^, VAL152 ^c^, ALA170 ^c^, ILE173 ^c^, VAL264 ^c^, LEU265 ^d^, LEU267 ^d^, LEU277 ^c^, LEU279 ^c^, LEU281 ^c^, ALA331 ^c^, ALA414 ^c^, ILE434 ^c^, ALA479 ^c^, VAL549 ^c^, VAL573 ^c^, VAL606 ^c^, LEU617 ^c^, ALA644 ^c^, LEU652 ^c^, VAL663 ^c^, VAL685 ^c^	(8 ^b^), TYR43 ^d^, TYR135 ^c^, TRP202 ^d^, TRP415 ^c^, TYR642 ^c^, PHE647 ^c^, PHE649 ^d^
**miR-2-BAMBI**	(38 ^a^), VAL42 ^c^, LEU45 ^d^, LEU46 ^c^, ALA47 ^c^, ALA50 ^c^, VAL58 ^c^, ALA111 ^c^, LEU132 ^c^, VAL264 ^c^, LEU265 ^d^, LEU267 ^d^, LEU277 ^c^, ALA331 ^c^, ALA414 ^c^, LEU435 ^c^, LEU439 ^c^, ALA479 ^c^, VAL549 ^c^, ALA648 ^c^, VAL663 ^c^.	(12 ^b^), TYR43 ^d^, TRP202 ^d^, TRP415 ^c^, TRP447 ^c^, PHE487 ^c^, PHE610 ^c^, PHE647 ^c^, PHE649 ^d^
**miR-3-SOD2**	(24 ^a^), VAL42 ^c^, LEU45 ^d^, LEU46 ^c^, ALA47 ^c^, VAL58 ^c^, ALA111 ^c^, LEU132 ^c^, ALA133 ^c^, VAL152 ^c^, ALA170 ^c^, ILE173 ^c^, LEU265 ^d^, LEU267 ^d^, LEU279 ^c^, LEU281 ^c^, VAL606 ^c^, LEU617 ^c^, ALA648 ^c^, LEU652 ^c^	(9 ^b^), TYR43 ^d^, TYR171 ^c^, TRP202 ^d^, TRP415 ^c^, PHE487 ^c^, PHE647 ^c^, PHE649 ^d^
**miR-4-BARD1**	(23 ^a^), VAL42 ^c^, LEU45 ^d^, VAL147 ^c^, VAL152 ^c^, ALA170 ^c^, ILE173 ^c^, VAL264 ^c^, LEU265 ^d^, LEU267 ^d^, LEU277 ^c^, ALA278 ^c^, LEU279 ^c^, LEU281 ^c^, ILE434 ^c^, LEU435 ^c^, ALA450 ^c^, VAL573 ^c^, VAL606 ^c^, ALA644 ^c^, ALA648 ^c^, LEU652 ^c^, VAL685 ^c^	(10 ^b^), TYR43 ^d^, TYR135 ^c^, TYR171 ^c^, TRP202 ^d^, TRP447 ^c^, PHE487 ^c^, PHE610 ^c^, TYR642 ^c^, PHE649 ^d^
**miR-5-TP53**	(30 ^a^), LEU45 ^d^, ALA47 ^c^, ALA50 ^c^, VAL58 ^c^, ALA111 ^c^, LEU132 ^c^, ALA133 ^c^, VAL147 ^c^, ALA151 ^c^, VAL152 ^c^, ALA170 ^c^, ILE173 ^c^, VAL264 ^c^, LEU265 ^d^, LEU267 ^d^, LEU277 ^c^, ALA278 ^c^, LEU279 ^c^, LEU281 ^c^, ILE434 ^c^, LEU435 ^c^, LEU439 ^c^, ALA450 ^c^, VAL606 ^c^, LEU617 ^c^, ALA644 ^c^, ALA648 ^c^, LEU652 ^c^, VAL685 ^c^	(10 ^b^), TYR43 ^d^, TYR135 ^c^, TYR171 ^c^, TRP202 ^d^, TRP447 ^c^, PHE610 ^c^, TYR642 ^c^, PHE647 ^c^, PHE649 ^d^

AA- amino acid; ^a^ Total hydrophobic residual amino acid involved in docking interaction; ^b^ Total aromatic ring containing amino acid residues with aromatic rings; ^c^ The residual amino acid of the receptor involved in interaction common to more than one complex interaction; ^d^ The residual amino acids of the receptor protein common to all the microRNA-mRNA binding to AGO.

## Data Availability

The datasets and the clinical data were obtained from specific databases as described in the methodology.

## Abbreviations

AGO	Argonaute (Receptor)
miRNA	microRNA
mRNA	Target genes
AA	Amino acid
H-bond	Hydrogen bond
PDB	protein data bank
RISC	RNA induced silencing complex
NA	Nucleic acid
BE	Binding energy
MFE	Minimum folding energy
